# Detection and molecular characterization of bovine enterovirus E2 from dairy calves with respiratory disease in Urumqi, Xinjiang, China

**DOI:** 10.3389/fcimb.2026.1800707

**Published:** 2026-06-29

**Authors:** Xintong Chen, Juanjuan Pan, Yangyuhua Wang, Yong Wei, Xiuyu Zhang, Haichun Jiang, Lei Zhang, Guiling Wu, Baoyu Chen, Jinxin Xie, Panpan Tong

**Affiliations:** 1 Laboratory of Animal Etiology and Epidemiology, College of Veterinary Medicine, Xinjiang Agricultural University, Urumqi, China; 2 Xinjiang Tianrun Beiting Animal Husbandry Co., Ltd, Changji, China; 3 Dahe Town Agricultural (Livestock) Development Service Center, Barkun County, Hami, China; 4 Aksu Regional Animal Disease Control and Diagnostic Center, Aksu, China; 5 Guangzhou Kexiang Biological Technology Co., Ltd, Guangzhou, China; 6 Xinjiang Key Laboratory of New Drug Research and Development for Herbivores, Urumqi, China

**Keywords:** bovine enterovirus, dairy calves, E2 subtype, outbreak, respiratory disease

## Abstract

**Introduction:**

Bovine enterovirus (BEV) is a contagious viral agent that can cause respiratory infections and disease outbreaks among calves. This study reports an outbreak that occurred in a population of dairy calves in northern Xinjiang in November 2024.

**Methods:**

Nasal swab samples were collected from 58 clinically symptomatic calves and analyzed for some bovine respiratory viruses using RT-PCR and viral metagenomic sequencing.

**Results:**

Viral metagenomic analysis annotated only one bovine pathogen, BEV, in respiratory disease samples. RT-PCR further confirmed that BEV was detected in all nasal swab samples from symptomatic dairy calves, while it was not detected in samples from healthy dairy cattle, suggesting that BEV may be the etiological agent of this respiratory disease. One BEV strain, designated XJ-FHT, was successfully isolated and found to be responsible for respiratory illness in calves. Comparative analysis of the whole genome, the encoded polyprotein, and the nucleotide and amino acid sequences of VP1 and P1, along with phylogenetic analysis of VP1 amino acid sequences, classified this isolate as belonging to the E2 subtype.

**Discussion:**

This study provides the first identification of a BEV-associated respiratory disease among calves in Xinjiang, China, in 2024. Molecular characterization and phylogenetic analysis identified the isolated strain as belonging to the E2 subtype. These findings highlight the potential role of BEV in bovine respiratory infections and emphasize the need for continued surveillance and preventive measures.

## Introduction

Enteroviruses (EVs) belong to the genus *Enterovirus* under the family *Picornaviridae* and include 12 recognized enterovirus species (A–L) along with three rhinovirus species (A–C) (Wu et al., 2025; Chang et al., 2024). Enterovirus E (EV-E) is a positive-sense, single-stranded RNA virus, approximately 20-30 nm in diameter, classified as bovine enterovirus 1 (BEV-1) in the Ninth Report of the International Committee on Taxonomy of Viruses (ICTV) (Zell et al., 2017). Its genome is approximately 7,100 to 7,450 nucleotides long, comprises a single open reading frame (ORF) bordered by untranslated regions (UTRs) at both the 5′ and 3′ ends. The ORF translates into a polyprotein precursor that undergoes proteolytic cleavage to yield four structural proteins (VP1, VP2, VP3, and VP4) and seven nonstructural proteins (2A, 2B, 2C, 3A, 3B, 3C, and 3D) (Chang et al., 2024; Zell et al., 2017). Sequence analysis of the VP1 gene has led to the identification of five EV-E subtypes, designated EV-E1 through EV-E5 (Wu et al., 2025; Chang et al., 2024).

EV-E has been reported in cattle across multiple regions, including China, the United States, Canada, Turkey, Ethiopia, and Brazil (Wu et al., 2025; Chang et al., 2024; Zell et al., 2017; Zhang et al., 2014; Zhu et al., 2014; Feng et al., 2025; Ji et al., 2022; Luo et al., 2023; Zhu et al., 2024; Chen et al., 2023, 2024; Rahpaya et al., 2018; Zhang et al., 2021; Faleye et al., 2018; Mosena et al., 2022; Tsuchiaka et al., 2017; McCarthy et al., 1999; Zell et al., 2006; Medina et al., 2024). This virus is not strictly host-specific, as it has also been detected in horses, goats, sheep, geese, opossums, and deer (Wu et al., 2025). Despite its broad host range, the pathogenic mechanisms and the virulence of EV-E in animals remain poorly characterized. EV-E has been identified in both healthy animals and those displaying symptoms such as pneumonia, diarrhea, and abortion (Wu et al., 2025). With the growing number of EV-E isolates obtained from diseased animals, interest in understanding their pathogenic potential has increased significantly.

In November 2024, an acute respiratory illness was reported in a dairy farm in Urumqi, Northern Xinjiang, China, affecting the entire cohort of dairy calves (n = 400; age < 6 months). Affected calves showed clinical signs of fever (> 40 °C), cough, purulent nasal discharge, anorexia, and weight loss. Treatment consisted of intramuscular administration of Florfenicol, Flunixin Meglumine, Penicillin, and Dipyrone, resulting in full recovery within approximately 7 days. The farm maintained a total of 1,724 dairy cattle, including 1,324 animals older than six months. Established in 2016, the farm sourced all cattle from Australia and routinely vaccinated calves against bovine viral diarrhea and infectious bovine rhinotracheitis.

The study aimed to identify the viral cause of a respiratory disease outbreak in cattle from Xinjiang, China, and to characterize the isolated bovine enterovirus strain through molecular and genomic analyses.

## Materials and methods

### Sample collection

In November 2024, during a respiratory disease outbreak in a dairy farm of Urumqi, Northern Xinjiang, China, nasal swab samples were collected weekly for four weeks from a total of 58 calves. In addition, nasal swab samples were collected from 36 clinically healthy cattle in adjacent pens of this farm. Each sample was collected and placed into a tube containing 1.5 mL phosphate buffer and stored at −80 °C by the farm veterinarian in accordance with approved procedures.

### Viral metagenomic analysis

A viral DNA library was constructed from pooled nasal swab samples using a previously described protocol (Xie et al., 2020). Briefly, 58 samples were combined, concentrated by ultracentrifugation, and subjected to viral nucleic acid extraction. Total viral nucleic acids were reverse transcribed, followed by random amplification. The resulting DNA products were purified and sequenced using the Illumina NovaSeq 6000 platform (Novogene, Tianjin, China), generating approximately 6 Gb of data per library, sufficient for viral screening.

Viral reads were extracted using BWA, Samtools, and Bedtools software. The virus-related reads were assembled into contigs using Trinity (Xie et al., 2020) and annotated via BLASTX against the NCBI-NR protein database to identify viral genomic sequences.

### BEV detection

Viral RNA was isolated from each nasal swab sample (200 µL) using the Geneaid extraction kit according to the manufacturer’s protocol. cDNA synthesis was performed with PrimeScript II Reverse Transcriptase (Takara, China) using the thermal conditions of 65 °C for 5 min, 42 °C for 60 min, and 95 °C for 5 min. PCR amplification of the BEV 5′-UTR and VP1 genes was performed using specific primers designed with Primer 5.0 software (**Table 1**), based on the sequence of the BEV-E2 reference strain IS1/Bos taurus/JPN/1990 (GenBank accession no. LC150009). The reactions were carried out with 2× TransStart^®^ FastPfu Fly PCR SuperMix (TransGen Biotech, Beijing, China) under the following thermal profile: an initial denaturation at a temperature of 95 °C for 2 min to ensure complete strand separation of the template, followed by 35 amplification cycles. Each cycle consisted of denaturation at 95 °C for 20 seconds (s) to separate double-stranded DNA, annealing at 52 °C for 20 s to allow primer binding, and extension at 72 °C for 30s to facilitate DNA synthesis by the polymerase enzyme. The reaction concluded with a final extension at 72 °C for 5 min to ensure complete elongation of all amplified fragments. Analysis of the amplified products was carried out through electrophoresis using an agarose gel (1%).

**Table 1:** Primers for BEV-E2 detection.

Primer	Primer sequence (5’-3’)	Product size (bp)	References
BEV-E2 5′-UTR F	GCGTGTCCTCGGGTTCACTTTC	403	LC150009
BEV-E2 5′-UTR R	CAGCGGTGTTTCTGCTCATTTG		
BEV-E2 VP1 F	TCGACCCACTCAATAGCTACAGAC	300	
BEV-E2 VP1 R	GGCAACAATGGTAAACTCAACATC		

### Cell culture, virus isolation, and electron microscopy

One randomly selected sample from the 58 BEV-positive samples was filtered through a 0.22 μm membrane and incubated with a monolayer of MDBK cells (approximately 70% confluence; provided by Tu Changchun’s laboratory) for 2 h at 37 °C in a 5% CO_2_ atmosphere to allow viral infection. After incubation, the inoculum was removed, and the cells were cultured in DMEM supplemented with 2% FBS and 1% penicillin–streptomycin. The cultures were incubated for 72 h under these conditions, after which the cells were subjected to three consecutive freeze–thaw cycles to release intracellular viral particles. To increase viral propagation, six additional cell passages were conducted. For transmission electron microscopy, purification of the viral particles was first carried out by sucrose density gradient centrifugation, followed by their negative staining by phosphotungstic acid (2%).

### Amplification and sequencing of the complete genome

PCR assays were designed to amplify fragments of the isolated strain genome using specific primer sets listed in **Table 2**. Viral RNA was extracted and subjected to reverse transcription-PCR (RT-PCR) according to the previously described BEV detection procedure. Amplified products yielding positive results were inserted into the pESI-T vector (Yeasen Biotech) and introduced into *E. coli* DH5α competent cells (Weidi Biotech) through chemical transformation. Three separate colonies from each PCR amplicon were chosen for Sanger sequencing, which was performed by Sangon Biotech (Shanghai, China).

**Table 2:** Primers for amplification of the complete genome sequence.

Primer	Primer sequence (5’-3’)	Product size (bp)	References
BEV-E2 1F	GTTGTACCCACCCCTGGG	1261	LC150009
BEV-E2 1261R	GCGTCAGGTAGCTTCCAGTACC		
BEV-E2 1139 F	CCACGACGCAACTTCAGTG	1177	
BEV-E2 2315R	GTACTGATAGTTGAGGGCGTCGTCC		
BEV-E2 2231F	GTCTTCCATTACTCTAGTGATACC	1226	
BEV-E2 3456R	CATCAACCCTGGTGACGAGGAGG		
BEV-E2 3402F	GAAAACGAGGTCTGGCAATCGTATC	1263	
BEV-E2 4664R	CGTGAAAGGTGCTGTTGACACC		
BEV-E2 4636F	GTCAAATGGTGTCAACAGCACC	1257	
BEV-E2 5897R	GTGCACCATTTCCTCCTACATGG		
BEV-E2 5768F	CCTAGCAGGTAGACCCACTCACAG	725	
BEV-E2 6492R	CAGAGTCATTCAAGCTCGATGCTTC		
BEV-E2 6351F	GTCACCAAGATGCAAGAGTGCATTG	1041	
BEV-E2 7391R	ACCCCATCCGGTGGGTGTATTC		

### Phylogenetic analyses

**Figure 1:**
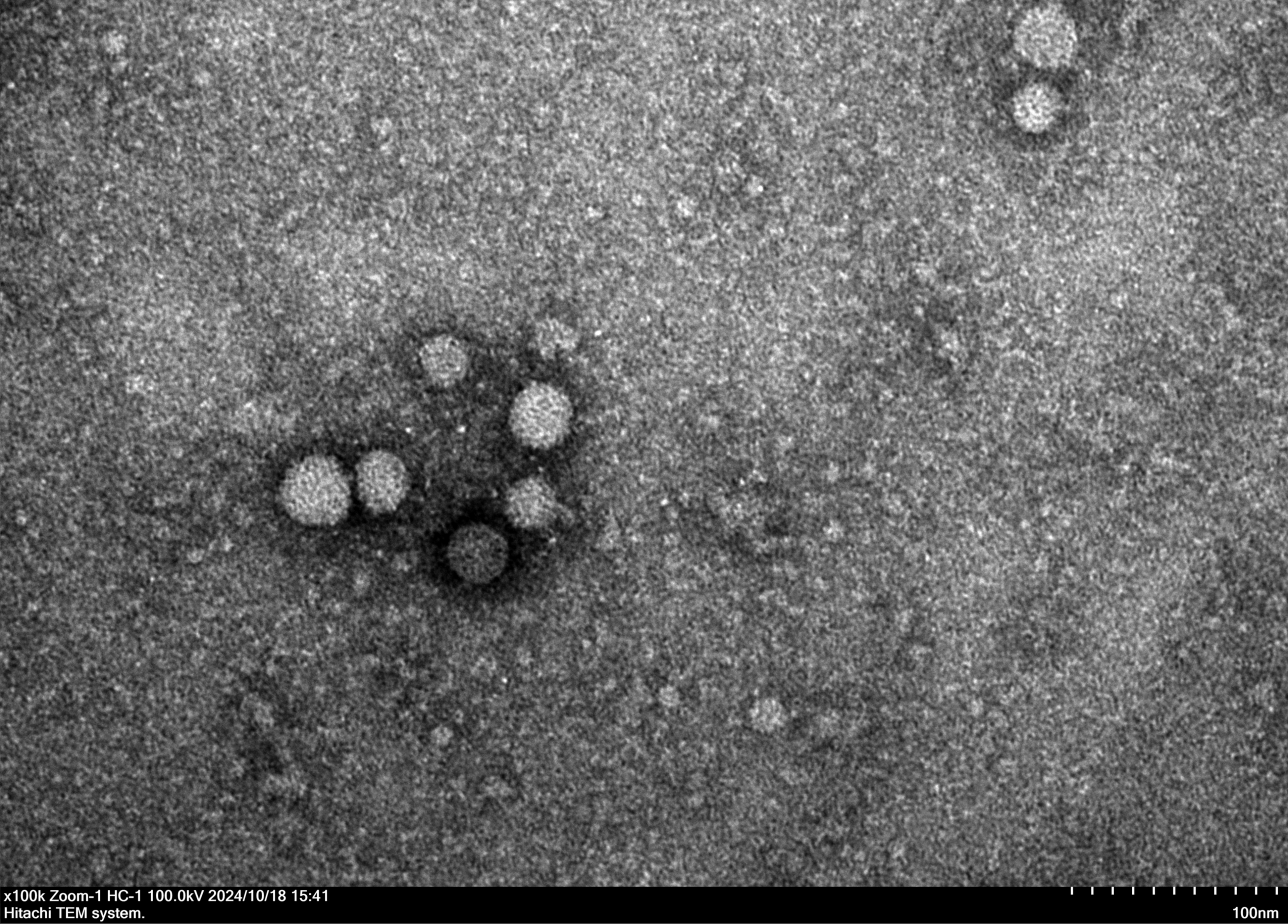
The virions of the BEV strain XJ-FHT display distinct morphological characteristics.

Eighteen BEV reference strains displaying various genotypes from China, Japan, the United States, Germany, Italy, and other regions were obtained from the GenBank database. Details of the reference sequences, along with their corresponding GenBank accession numbers, are presented in **Figure 1**. Sequence similarity was analyzed using MegAlign software (Lasergene v7.1). Phylogenetic analysis of the VP1 gene was performed using the maximum-likelihood method with the Tamura–Nei substitution model. The robustness of the phylogenetic tree was assessed by bootstrap testing with 1,000 replicates (Kumar et al., 2016).

## Results

### Overview of the virome

Metagenomic analysis of 58 nasal swab samples collected from calves showing respiratory disease on a dairy farm in Urumqi, Xinjiang, yielded 1,701,636 sequencing reads. Among these, 56,154 reads (3.3%) were annotated as mammalian viral sequences, including BEV, retroviruses, and influenza C virus. Of the mammalian viral reads, 39,970 were 150 nucleotides (nt) in length and displayed 75.5 – 78.9% nt identity with various genes of BEV-E2 reference strain BEV IS1/Bos taurus/JPN/1990 (GenBank accession no. LC150009).

### Viral detection

Initial RT-PCR analysis targeting both the 5′-UTR and VP1 genes confirmed BEV in nasal swab samples from all 58 dairy calves displaying respiratory symptoms. However, BEV was not detected in nasal swab samples from 36 healthy dairy cattle. Other some bovine respiratory viral pathogens, including bovine viral diarrhea virus (BVDV), bovine coronavirus (BCV), and bovine herpesvirus type 1 (BHV-1), were not detected in these samples. These findings indicate that BEV was the likely causative agent of the respiratory disease outbreak in calves in northern Xinjiang, China.

### BEV isolation

Viral isolation was conducted using a nasal swab sample obtained from a symptomatic calf that tested positive for BEV. MDBK cells were used for virus propagation, and distinct CPEs were observed as early as the first passage. PCR analysis confirmed the successful isolation of the BEV strain (XJ-FHT) after seven passages. Compared with mock-infected controls, BEV-infected MDBK cells displayed rounding within 24 h post-infection. Transmission electron microscopy demonstrated that viral particles from the BEV XJ-FHT isolate displayed the characteristic morphology of BEV, with virions measuring approximately 20–30 nm in diameter (**Figure 1**).

### Sequencing and analysis of the whole genome of the BEV XJ-FHT strain

To determine the whole-genome sequence of the BEV XJ-FHT strain, a set of specifically designed primers was used to amplify overlapping regions of the viral genome (**Table 2**). The amplified fragments were sequenced, and the overlapping sequences were assembled to reconstruct the full-length viral genome. The analysis of the sequence revealed that the complete genome of the BEV XJ-FHT strain comprised 7,391 nt (accession number: PQ469036), including an 800-nt 5′ UTR, a 6,525-nt polyprotein-coding region, and a 66-nt 3′ UTR. The ORF spanned nt 801 to 7,325 and encoded a predicted polyprotein consisting of 2,174 amino acid (aa) residues.

### The BEV XJ-FHT strain shared high sequence identity with EV-E2 reference strains

Comparative genomic analysis was carried out to assess the genetic relationship between BEV XJ-FHT and representative reference BEV strains. Complete genome sequences of BEV strains available in the GenBank database were retrieved for alignment. The results showed that BEV XJ-FHT shared 66.6–81.3% nt sequence identity with other BEV strains, representing the highest similarity (81.3%) with the BEV-E2 reference strain BEV IS1/Bos taurus/JPN/1990 (**Table 3**).

**Table 3:** Percent identity of nucleotide and amino acid sequences between XJ-FHT isolated strain and bovine enterovirus reference strains.

Strains	GenBank Accession No.	XJ-FHT Identity (%)						Subgenotype
		Complete Genome	VP1		P1		Polyprotein	
			nt	aa	nt	aa	aa	
BEV IS1/Bos taurus/JPN/1990	LC150009	81.3	78.9	94.3	79.9	95.7	96.8	E2
K2577	AF123432	80.4	77.0	94.3	77.4	94.6	96.4	E2
PS/42	DQ092792	77.7	73.2	90.7	75.9	93.1	94.4	E2
EV_NGR_2017	MH719217	77.6	74.8	90.7	75.8	92.6	94.3	E2
UFSM-SV89	OL660538	79.5	77.7	92.9	78.0	94.8	96.3	E2
VG-5-27	D00214	78.1	69.8	77.6	73.3	86.6	92.5	E1
LC-R4	DQ092769	78.5	69.3	76.9	73.5	86.3	93.1	E1
HY12	KF748290	79.1	70.6	81.1	72.9	87.4	92.8	E3
JL-DH13	MN598020	79.6	72.2	81.5	73.8	88.0	93.1	E3
PAK_NIH_92E1	JQ690746	–	70.9	83.5	–	–	–	E4
PAK_NIH_123E1	JQ690747	–	70.8	83.5	–	–	–	E4
MexKSU/5	KU172420	73.0	52.6	52.3	60.9	65.2	82.9	E5
GX20-1	MW477470	72.5	53.2	52.3	61.0	64.9	82.8	E5
JS20-1	MW579538	72.5	53.2	52.3	61.0	64.9	82.8	E5
BEV-261	DQ092770	67.2	56.9	53.8	62.2	66.3	72.8	F1
PS-89	DQ092795	67.8	57.5	54.4	63.2	66.3	73.3	F2
PS87/Belfast	DQ092794	66.6	56.5	54.4	61.4	65.0	72.8	F3
W1	AY462106	67.1	55.2	52.6	61.8	65.3	72.9	F4

AA sequence analysis of the encoded polyprotein was also conducted. The BEV XJ-FHT strain displayed high similarity with BEV-E2 reference strains, showing 94.3 – 96.8% identity with strains including BEV IS1/Bos taurus/JPN/1990, K2577, PS/42, EV_NGR_2017, and UFSM-SV89 (**Table 3**). To evaluate the sequence similarity between BEV XJ-FHT and reference strains, nt and aa sequences of VP1 and P1 were analyzed. As shown in **Table 3**, the VP1 region of XJ-FHT shared 73.2 – 78.9% nt and 90.7 – 94.3% aa identity with BEV-E2 reference strains, including BEV IS1/Bos taurus/JPN/1990, indicating that the XJ-FHT isolate is evolutionarily most closely related to BEV-E2 strains. As presented in **Table 3**, the P1 region of the XJ-FHT strain showed 75.8 – 79.9% nt identity and 92.6 – 95.7% AA identity with BEV-E2 reference strains, including BEV IS1/Bos taurus/JPN/1990, confirming that XJ-FHT belongs to the BEV-E2 genotype. These results indicate that the XJ-FHT isolate is closely related to BEV-E2 strains, as determined by sequence comparisons.

### Phylogenetic analysis

**Figure 2:**
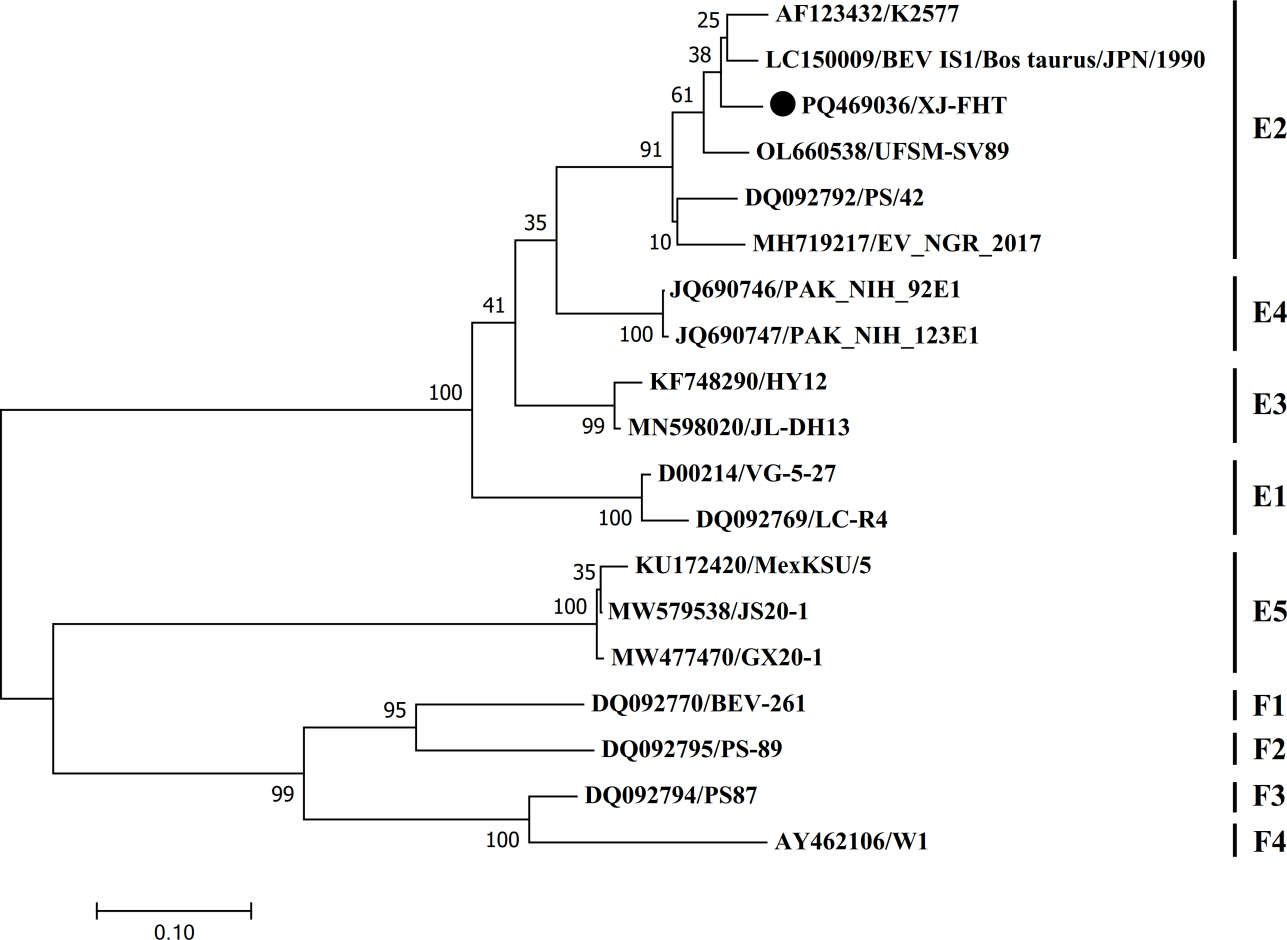
Phylogenetic analysis was performed using the VP1 aa sequences of the newly isolated strain and 18 reference BEV strains. Sequence alignment was conducted, followed by phylogenetic tree construction using the maximum-likelihood (ML) method in MEGA7 software with 1,000 bootstrap replicates. The BEV XJ-FHT strain isolated in this study is indicated by fixed circles in the phylogenetic tree. VP1 aa sequences of all reference BEV strains were retrieved from the GenBank database.

Phylogenetic analysis of VP1 AA sequences demonstrated that XJ-FHT clusters closely with established BEV-E2 reference strains, including BEV IS1/Bos taurus/JPN/1990, K2577, PS/42, EV_NGR_2017, and UFSM-SV89. As shown in **Figure 2**, XJ-FHT groups within the BEV-E2 lineage, confirming its close genetic relationship with these strains and validating its classification as a BEV-E2 isolate.

## Discussion

Calf respiratory disease not only results in acute mortality and increased treatment expenses but also significantly compromises the animal’s future productivity. By generating chronically debilitated cattle that serve as persistent sources of infection, the disease presents an ongoing risk to herd health, productivity, and overall management efficiency. The affected calves in this study exhibited clinical signs such as fever (> 40 °C), coughing, purulent nasal discharge, anorexia, and weight loss, resulting in significant economic losses for the dairy farm in Xinjiang.

Previous studies have reported BVDV, BCV, and BHV-1 as the primary etiological agents of respiratory infections in cattle (Zhu et al., 2024; Chen et al., 2023, 2024). However, some of them were not detected in nasal swab samples collected during the November 2024 outbreak. This may be due to the small sample size in this study; therefore, future work will include a larger sample size. Additionally, *Mannheimia haemolytica*, *Pasteurella multocida*, and *Mycoplasma bovis* are known bovine respiratory pathogens that were not tested in this study. Thus, these pathogens should be included in subsequent experiments to comprehensively assess their potential role in the outbreak.

Previous studies conducted in other countries have demonstrated that BEV-E2 is a viral pathogen capable of inducing respiratory disease in cattle (Rahpaya et al., 2018; Zhang et al., 2021). In the present study, virome and RT-PCR analysis confirmed that all nasal swab samples from respiratory symptomatic calves were only positive for BEV-E2, whereas healthy cattle in adjacent pens were virus-negative, thereby identifying it as the likely etiological agent of the respiratory disease. These results strongly suggest that BEV-E2 was responsible for the respiratory outbreak among calves on the dairy farm in Urumqi, Xinjiang. This study reports the first detection of BEV in nasal swabs from cattle in China, supporting the proposed role of this virus in bovine respiratory illness. To date, five BEV subtypes (E1–E5) have been identified, and these viruses are distributed globally among cattle populations, including those in China and the United States (Wu et al., 2025; Chang et al., 2024; Zell et al., 2017; Zhang et al., 2014; Zhu et al., 2014; Feng et al., 2025; Ji et al., 2022; Luo et al., 2023; Zhu et al., 2024; Chen et al., 2023, 2024; Rahpaya et al., 2018; Zhang et al., 2021; Faleye et al., 2018; Mosena et al., 2022; Tsuchiaka et al., 2017; McCarthy et al., 1999; Zell et al., 2006; Medina et al., 2024). Among them, only the pathogenicity of BEV-E2 has been well established, as it is known to cause respiratory disease along with diarrhea and abortion in cattle (Rahpaya et al., 2018). In China, BEV-E2 infection has emerged as a novel infectious disease within the past fifteen years, posing a serious risk to the sustainable development and health of the cattle industry (Wu et al., 2025; Chang et al., 2024; Zhang et al., 2014; Feng et al., 2025; Luo et al., 2023). Chinese BEV-E2 strains have been isolated and identified from calves and lactating cows showing diarrhea in several regions, including Inner Mongolia, Shandong, Beijing, Jilin, Guangdong, Guangxi, and Xinjiang (Wu et al., 2025; Chang et al., 2024; Zhang et al., 2014; Feng et al., 2025; Luo et al., 2023). In the present study, sequence characterization and phylogenetic analyses showed that the XJ-FHT isolate represented the highest similarity to the reference strain BEV IS1/Bos taurus/JPN/1990 and to BEV-E2 strains previously associated with respiratory disease in Chinese calves. These findings highlight the need for increased surveillance and further investigation into the role of BEV in bovine respiratory infections. At present, no commercial BEV vaccine is available in China. In this study, BEV-E2 was identified as a causative agent of respiratory disease and, together with strains previously associated with diarrhea (Wu et al., 2025; Chang et al., 2024; Zhang et al., 2014; Feng et al., 2025; Luo et al., 2023), will be used to develop an inactivated BEV-E2 vaccine. This vaccine is intended to prevent both respiratory and diarrheal infections in cattle in China.

## Conclusion

In conclusion, BEV-E2 was only detected in all nasal swab samples from 58 calves with respiratory symptoms but not in healthy controls, suggesting it was the probable causative agent of the 2024 respiratory disease in northern Xinjiang. These findings highlight the potential role of BEV-E2 in bovine respiratory illness in China and underscore the importance of developing an effective vaccine to control its transmission and mitigate disease severity.

## Data Availability

The data presented in the study are deposited in the GenBank repository, accession number PQ469036.
